# The diagnostic and predictive accuracy of the PRISMA-7 screening tool for frailty in older adults: A systematic review protocol

**DOI:** 10.12688/hrbopenres.13042.1

**Published:** 2020-05-22

**Authors:** Owen Higginbotham, Aoife O'Neill, Louise Barry, Aoife Leahy, Katie Robinson, Margaret O'Connor, Rose Galvin

**Affiliations:** 1 School of Allied Health, University of Limerick, Limerick, Co Limerick, V94 T9PX, Ireland; 2 Department of Geriatric Medicine, University Hospital Limerick, Limerick, Co. Limerick, V94 F858, Ireland

**Keywords:** PRISMA-7, Frailty, older adults, diagnostic accuracy, predictive accuracy, sensitivity and specificity, review

## Abstract

**Background: **Older adults are at risk of adverse outcomes due to frailty. A number of frailty screening instruments have been developed to identify older adults at increased risk of frailty. This systematic review and meta-analysis will look to examine the diagnostic accuracy of the Program of Research to Integrate the Services for the Maintenance of Autonomy 7 (PRISMA-7).

**Methods and analysis: **A systematic literature search will be conducted from 2008-February 2020 in PubMed, EMBASE, CINAHL, EBSCO and the Cochrane Library to identify validation studies of the PRISMA-7 tool.  A pre-specified PRISMA-7 score of ≥3 (maximum score 7 points) will be used to identify frailty in older adults. Prospective or retrospective cohort studies, cross-sectional studies and the control arm of randomised controlled trials will be included that attempt to validate the diagnostic accuracy of the PRISMA-7 screening tool in older adults across all healthcare settings when compared to a reference standard. The predictive accuracy of the PRISMA-7 tool will also be explored. Study quality will be assessed by the QUADAS-2 tool. A bivariate random effects model will be used to generate pooled estimates of sensitivity and specificity. Statistical heterogeneity will be explored using validated methods.

**Ethics and dissemination: **Formal ethical approval is not required as primary data will not be collected. The results will be disseminated through a peer-reviewed publication, conference presentation and the popular press.

**Protocol registration: **Awaiting registration with the International Prospective Register for Systematic Reviews (PROSPERO).

## Introduction

Global demographic trends suggest that the number of adults aged 65 years or older will more than double by 2050 ^1^, resulting in an increased burden on healthcare systems and a disproportionate increase in the burden on emergency departments (EDs). Older people are frequent users of the healthcare system ^2^, and they are more likely to experience adverse health outcomes including functional decline, unplanned ED presentation and hospitalisation following emergency care ^3^. Chronological age alone cannot reliably predict health service utilisation, similarly level of disability or multi-morbidity cannot exclusively explain healthcare service utilisation ^2, 4^. Increased utilisation of healthcare services is in part due to increased frailty ^5^.

The concept of frailty has evolved over the years. In 2001, Fried and Walston described a frailty phenotype with five related components to form a cycle of frailty: motor weakness, slowness, exhaustion, low activity and weight loss ^6^. At the same time, the deficit accumulation model was also proposed, which included the number of diseases, conditions, and co-morbidities across many domains to determine frailty status ^7^. Frailty has recently been defined by a consensus group as a medical syndrome with “multiple causes and contributors which is characterised by diminished strength, endurance, and reduced physiologic function that increases an individual’s vulnerability for developing increased dependency and/or death” ^8^. In a systematic review of 21 community-based studies of older adults, the prevalence of frailty was reported to range from 4% to 59.1% ^9^. Frailty is over-represented among acute hospital admissions, with 24–40% of older adults presenting with moderate to severe frailty ^10^. The wide range of prevalence rates possibly reflect variation across the studies in terms of definitions of frailty and methodological approaches.

Regardless of the definition used, the presence of frailty has been shown to predict mortality ^11, 12^, increased risk of falls ^13^, depressive symptoms ^14^, disability ^15^, dementia ^16^, delirium ^17^, decrease in ability to carry out activities of daily living (ADLs) ^18^, reduced quality of life and functional impairment ^19^, use of healthcare services ^2^, and institutionalisation ^20^. Recent evidence proposes that while frailty may be age related, it is not age dependant. Hanlon *et al*. (2018) report that frailty and pre-frailty are associated with female sex, obesity and underweight, smoking, socioeconomic deprivation, and multimorbidity ^21^.

In recent years, a number of frailty screening tools have been developed and have demonstrated an increased risk of adverse outcomes in frail older adults ^22^. These frailty screening tools broadly focus on physical markers of decline or on the accumulation of deficits in physical, cognitive, mental health and functional domains ^23^. One such screening tool is the PRISMA-7 (Program of Research to Integrate the Services for the Maintenance of Autonomy 7). The PRISMA-7 was derived in Canada in 2007 and comprises a brief, 7-item yes/no questionnaire where a cut off score of ≥3 is used to identify frail older adults. The questions cover age, general health, activities and social supports, with each answer receiving a score of one or zero ^24^. The PRISMA-7 is recommended by the British Geriatric Society (2014) ^25^ as a quick and simple frailty screening tool. It is also recommended for use by the Asia-Pacific Clinical Practice Guidelines for the Management of Frailty (2017) ^26^, and by the National Institute for Health and Care Excellence (NICE) guidelines (2016) ^27^. Since its derivation, a number of studies have attempted to examine its diagnostic accuracy identifying frailty in older adults and its predictive accuracy in determining risk of adverse outcomes among those classified as frail ^28, 29^. This systematic review aims to synthesise the totality of evidence regarding the diagnostic and predictive accuracy of the PRISMA-7 at identifying frailty in older adults and subsequent risk of adverse outcomes.

## Methods and analysis

### Study design

A systematic review will be conducted to identify studies that have attempted to validate the PRISMA-7 screening tool in older adults, across healthcare settings. The systematic review will conform to the principles outlined in the Cochrane Handbook for Systematic Reviews of Diagnostic Test Accuracy ^30^ and the Preferred Reporting Items for Systematic Reviews and Meta-Analyses Protocols (PRISMA) standardised reporting guidelines will be referenced ^31^. The Preferred Reporting Items for Systematic Reviews and Meta-Analyses Protocols (PRISMA-P) guidelines were used in completing this review protocol ^32^; see *Reporting guidelines* ^33^. 

### Eligibility criteria

Studies will be selected using the population, experimental test, reference standard and study designs (PEOS) criteria. The population of interest include older adult’s ≥65 years (mean/median age ≥65 years) across healthcare settings where the PRISMA-7 screening tool was administered and compared to a reference standard. While there is no ‘gold standard’ for the definition of frailty, for the purposes of this review we will use five different reference standards including the Comprehensive Geriatric Assessment (CGA), clinical judgement by an expert panel, Fried Physical Frailty Phenotype, the Functional Autonomy Measurement System and the Frailty Index. The CGA is a multidisciplinary evaluation of multiple domains, including medical, mental and functional problems of older persons ^34^. Clinical judgement by an expert panel (including geriatricians, geriatric nurses and general practitioners) using validated screening tools to inform decision-making, including but not limited to tools including the Mini Mental State Exam (MMSE) ^35^, InterRAI-Community Health Assessment (InterRAI-CHA) ^36^, Identification of Seniors at Risk (ISAR) ^37^. The Functional Autonomy Measurement System (SMAF) ^38^ is a 29-item scale developed upon the concepts of health and disability as described by the World Health Organisation’s (WHO) ^39^. Fried *et al*. developed the Fried Physical Frailty Phenotype, which assesses frailty through five physical components; unintentional weight loss, self-reported exhaustion, weakness (grip strength), slow walking speed and low physical activity ^6^. The frailty index (FI) is a cumulative model of quantifying frailty, the model counts “deficits in health”, on the premise that the more deficits an individual has the greater probability of frailty. When a minimum of 30 variables are included, the FI is strongly associated with risk of death, institutionalisation and worsening health status ^40^.

Prospective or retrospective cohort studies or cross-sectional studies will be included where the PRISMA-7 is used to screen older adults and compared to one or more of these reference standards to explore the diagnostic accuracy of the tool. In terms of establishing the predictive accuracy of the PRISMA-7, the reference standard will comprise adverse short and long-term outcomes experienced post-administration of the PRISMA-7. Adverse outcomes will include functional decline, unplanned ED presentation, unscheduled hospital admission, admission to long-term care or mortality.

### Exclusion criteria

Studies will be excluded if their populations mean or median age is <65 and where data cannot be extracted separately on those ≥65 years. Grey literature will not be included.

### Information sources/search strategy

The search will include studies published from 2008 (year that the PRISMA-7 was derived) to the present and will be limited to the title, abstract, and index terms used to describe the article. A PubMed search strategy can be seen in Table 1.

**Table 1.** PubMed search strategy, modified accordingly for use in other databases.

Search	Search string	Entries
#1	((PRISMA Seven[Title/Abstract] OR PRISMA-7[Title/Abstract] OR PRISMA 7[Title/Abstract] OR (Program of Research on Integration of Services for the Maintenance of Autonomy Seven)[Title/Abstract] OR (Program of Research on Integration of Services for the Maintenance of Autonomy 7)[Title/Abstract])) AND ((older adult)[Title/Abstract] OR elderly[Title/Abstract] OR geriatric [Title/Abstract] OR aging[Title/Abstract] OR aged[Title/Abstract] OR senior [Title/Abstract] OR (older person)[Title/Abstract] OR (older people)[Title/Abstract] OR (aged 65[Title/Abstract] OR aged 65+)[Title/Abstract] OR (aged over sixty five)[Title/Abstract] OR retired[Title/Abstract])	141

The following electronic databases will be searched:

• PubMed

• EMBASE

• CINAHL

• EBSCO and Cochrane Library

Studies in all languages will be included and translated. This search will be supplemented by hand searching references of retrieved papers and searching Google Scholar. All searches will be imported into Endnote reference management system and duplicates will be removed.

### Study selection and data extraction

Titles and abstracts will be independently screened for relevance based upon the above inclusion criteria by two reviewers (OH, AON). Studies deemed eligible for inclusion will be read fully and their suitability for inclusion will be independently determined by RG. Any disagreements will be managed by discussion. Data will be extracted from the included studies by two independent reviewers (AON, OH) using standardised forms that will include, study type and setting, patient demographics (age, gender) and clinical characteristics including relevant inclusion and exclusion criteria, person who administered the PRISMA-7. Any disagreements in data extraction will be resolved by discussion. If the disagreement persists, a third author (RG) will independently extract the data. If a study presents missing, unclear or incompletely reported data, we will attempt to contact the study authors to obtain the data. The extent of missing data will be documented in the extraction form.

### Risk of bias in individual studies

Methodological quality of the selected studies will be evaluated independently by two reviewers (OH and RG) using the QUADAS-2 tool ^41^, a validated tool for the quality assessment of diagnostic and prognostic accuracy studies. Disagreements will be resolved by a third reviewer (KR).

### Data synthesis and analysis

Statistical analysis will be completed using Stata version 12 (StataCorp, TX, USA) by RG. A series of 2 × 2 tables (PRISMA-7 ≥3) will be constructed and data will be populated on the number of true positives, false positives, true negatives and false negatives from each study. Authors of included studies will be contacted to provide additional data on study outcomes where necessary. Pooled estimates of sensitivity and specificity with 95% confidence intervals (95% CIs) will be calculated to determine the diagnostic and predictive accuracy of the PRISMA-7 using a bivariate random effects model. We have employed this methodology in previous studies ^42– 44^. In the context of diagnostic accuracy, sensitivity refers to the proportion of older adults who are correctly classified as frail (PRISMA-7 ≥3) when compared to the reference standard whereas specificity refers to those who are correctly classified as non-frail (PRISMA-7 <3). In terms of predictive accuracy, sensitivity refers to the proportion of frail older adults (PRISMA-7 ≥3) who experience an adverse outcome whereas specificity refers to those who are non-frail (PRISMA-7 <3) and do not experience a subsequent adverse outcome.

Individual and summary estimates of sensitivity and specificity will be graphed on a receiver-operating characteristic (ROC) graph. Statistical heterogeneity will be examined using the variance of logit-transformed sensitivity and specificity, with smaller values indicating less heterogeneity between studies. Bayes’ theorem will be applied to estimate the post-test probability of an adverse outcome ^45^. The *c* statistic, or area under the curve, with 95% CI will be used to represent model discrimination. Values between 0.7 and 0.9 indicate moderate accuracy and values greater than 0.9 demonstrate high accuracy ^46^. We will conduct sensitivity analyses to examine the impact of methodological quality, reference standard used and setting of care on the diagnostic and predictive value of the PRISMA-7 where possible. Funnel plots will be generated to examine publication bias.

## Discussion

Frailty is a dynamic condition that contributes to functional decline in older adults ^47^. Early identification of frailty can improve care for older adults and reduce the risk of exacerbation of pre-frail states ^48^. The British Geriatric Society recommends that all encounters between health and social care staff and older people include an assessment of frailty ^49^. Better identification of frailty or pre-frail individuals will allow for more specific and tailored interventions to be provided to these individuals. The European Innovation Partnership on Active and Healthy Ageing (EIP on AHA) action plan A3, states that successful prevention of frailty and functional decline requires more knowledge about the risk factors and the stratification of patients ^50^. The EIP on AHA recommend the use of short risk-prediction instruments, to identify individuals at risk of frailty ^50^. These instruments should be simple, valid, accurate and reliable ^23^. The PRISMA-7 ^24^ is a brief instrument used to identify frailty in older adults. Its use is recommended in International guidelines ^26, 27^. This systematic review will provide important information about the quantity and quality of studies validating the PRISMA-7. It will summarise the evidence regarding the diagnostic and predictive value of the PRISMA-7 at identifying adverse outcomes in frail older adults across a variety of settings.

## Dissemination

The systematic review will be published in a peer-reviewed journal and presented at appropriate conferences (e.g. Irish Gerontology Society Annual meeting, Health Research Board).

## Data availability

### Underlying data

No underlying data are associated with this article.

### Reporting guidelines

Figshare: PRISMA-P checklist for ‘The diagnostic and predictive accuracy of the PRISMA-7 screening tool for frailty in older adults: A systematic review protocol’. https://doi.org/10.6084/m9.figshare.12229125 ^33^.

The completed PRISMA-P checklist is available under the terms of the Creative Commons Zero “No rights reserved” data waiver (CC0 1.0 Public domain dedication).
