# Supplementary material for: The diagnostic and predictive accuracy of the PRISMA-7 screening tool for frailty in older adults: A systematic review protocol
Source: HRB Open Res. 2020 May 22;3:26. [Version 1] doi: 10.12688/hrbopenres.13042.1 (PMC8201424; doi:10.12688/hrbopenres.13042.1)
Supplement: Peer review history

# Peer review: Reviewer response for version 1

**DOI:** 10.21956/hrbopenres.14138.r29498

**Published:** 2021-06-11

**Authors:** Aisling O’Halloran

**Affiliations:** 1 The Irish Longitudinal Study on Ageing, Medical Gerontology, School of Medicine, Trinity College Dublin, Dublin, Ireland

In this manuscript the authors present a study protocol to conduct a systematic literature review and meta-analysis to validate the diagnostic and predictive accuracy of a screening tool, the Program of Research to Integrate the Services for the Maintenance of Autonomy 7 (PRISMA-7), for identifying frailty among older adults. The authors confirm that this study protocol has been submitted for registration with the PROSPERO - the International Prospective Register for Systematic Reviews.

 This is a well structured and described study protocol which adheres to the guidance for the conduct of systematic reviews and meta-analyses including the principles outlined in the Cochrane Handbook for Systematic Reviews of Diagnostic Test Accuracy and the Preferred Reporting Items for Systematic Reviews and Meta-Analyses Protocols (PRISMA-P). The study design, eligibility and exclusion criteria, and the data extraction, synthesis and analysis strategies are clear. The independent evaluation of selected studies for methodological quality and bias risk will be important. In addition to the main analyses the sensitivity analyses will be important to the overall interpretation and applicability of the main findings. 

 In summary, this is a robust study protocol on a very current topic in geriatric medicine, using biological age rather than chronological age to equitably allocate health care resources to older adults. I look forward with great interest to the publication of the evidence from this systematic review and meta-analysis investigating the diagnostic and predictive accuracy of the PRISMA-7 screening tool for frailty in older adults.

 I have outlined some minor points which the authors may consider below.

 Specific comments:

1. I would suggest including the meta-analysis component in the title e.g. The diagnostic and predictive accuracy of the PRISMA-7 screening tool for frailty in older adults: A systematic review and meta-analysis protocol [version 1; peer review: 1 approved]
2. Please also state that the sex/gender is also asked on item 2 ‘Are you male?’ on the PRISMA-7.  See Page 3, bottom of column 1 ‘The questions cover age, general health, activities and social supports, with each answer receiving a score of one or zero.’
3. On pages 3 and 4 the PRISMA-7 was stated to be derived in 2008 and 2007 respectively. From Ref 24 (Raîche et al, 2008), the correct year is 2008, which also reflects the systematic review period from 2008-2020.
4. Study selection and data extraction: It is important that the authors include (or explicitly state they will include) the reference standard measures of frailty (CGA, frailty phenotype, frailty index, etc) on the standardised forms along with study type and setting, patient demographics (age, gender) and clinical characteristics including relevant inclusion and exclusion criteria, person who administered the PRISMA-7.
5. As male sex provides an additional score of 1 on the PRISMA-7, do the authors have a strategy to look for or deal with any sex bias among the studies included in the systematic review or meta-analyses? It may be worth looking at sex as an additional sensitivity analysis along with methodological quality, reference standard used and setting of care on the diagnostic and predictive value of the PRISMA-7.

Is the study design appropriate for the research question?

Yes

Is the rationale for, and objectives of, the study clearly described?

Yes

Are sufficient details of the methods provided to allow replication by others?

Yes

Are the datasets clearly presented in a useable and accessible format?

Not applicable

Reviewer Expertise:

Frailty, biology of ageing, gerontology, health epidemiology.

I confirm that I have read this submission and believe that I have an appropriate level of expertise to confirm that it is of an acceptable scientific standard.

# Peer review: Reviewer response for version 1

**DOI:** 10.21956/hrbopenres.14138.r27440

**Published:** 2020-06-23

**Authors:** Maria Costello, Michelle Canavan

**Affiliations:** 1 HRB-Clinical Research Facility, National University of Ireland Galway, Galway, Ireland; 2 HRB-Clinical Research Facility, National University of Ireland, Galway, Ireland

This is a study protocol for a systematic review and meta-analysis which will explore the diagnostic and predictive accuracy of the PRISMA-7 tool for identifying frail older adults. This protocol is awaiting registration with the International Prospective Register for Systematic Reviews (PROSPERO). 

 Overall this was a well written protocol which adhered to the PRISMA guidelines for systematic reviews. Addressing the following minor comments may add value to the protocol.

1. We suggest the title be amended to include the word meta-analysis.
2. The abstract notes that the control arm of randomised controlled trials will be used in this review, however this is not stated within the inclusion criteria. Please specify further if such trials will be included within this study as it will impact interpretation of both risk of bias, study quality and analysis.
3. Important factors to consider in the inclusion criteria are timing of measurement of the PRISMA-7 and location of patient. It is noted that this review will include studies “across all health care settings”. We agree that this is an appropriate search term to ensure all relevant studies are included. It may be worthwhile further categorising health care settings included or excluded as there will be clinical difference between the PRISMA-7 measured early in the course of an acute hospital admission versus measurement within stable general practice setting. Both are very different settings for patient care and severity of illness can influence interpretation of frailty scales making timing of measurement, experienced rater and later reassessment essential to avoid inappropriate labelling in the context of being acutely unwell. It would be useful to have these established categories for further subgroup analysis.
4. It would be worthwhile mentioning how to deal with repeat measurements performed during the study and in the case of multiple measurements which time point measurement would be extracted.
5. It is noted that there are several comparators for the PRISMA-7 scale. We suggest the inclusion of a subgroup analysis based on comparator used i.e. clinical judgment by expert/panel of experts versus use of an additional index/scale. This is of clinical importance when choosing to incorporate tools that may be in lieu of traditional bedside impression and clinical evaluation.
6. We note that regarding the *c* statistic that values “greater than 0.9 demonstrate high accuracy and Values between 0.7 and 0.9 indicate moderate accuracy”. Many commonly used prediction tools in clinical practice have lower *c *statistic values e.g. CHADS2 score had a c-statistic value 0.637 compared to CHADS2-VASc at 0.647 ^1^ so it would be relevant to include in the discussion that tools still need to be user-friendly and simple to apply in the busy clinical setting which is an advantage of the PRISMA-7 compared to other more complex frailty tools.

Overall this is a very interesting study which focuses on answering an important clinical question for health care professionals in the planning of service provision for frail older adults across a variety of disciplines.

Is the study design appropriate for the research question?

Yes

Is the rationale for, and objectives of, the study clearly described?

Yes

Are sufficient details of the methods provided to allow replication by others?

Yes

Are the datasets clearly presented in a useable and accessible format?

Not applicable

Reviewer Expertise:

Geriatric medicine, neurocognitive syndromes, cognitive and functional decline.

We confirm that we have read this submission and believe that we have an appropriate level of expertise to confirm that it is of an acceptable scientific standard.

## References

[1] : Identifying patients at high risk for stroke despite anticoagulation: a comparison of contemporary stroke risk stratification schemes in an anticoagulated atrial fibrillation cohort. *Stroke* .2010;41(12) : 10.1161/STROKEAHA.110.590257 2731-8 10.1161/STROKEAHA.110.590257 20966417
